# 2497: Risk factors for poor retention in HIV care using clinic and statewide surveillance data

**DOI:** 10.1017/cts.2017.286

**Published:** 2018-05-10

**Authors:** Rebecca Duron, Michael Mugavero, Andrew Westfall

**Affiliations:** University of Alabama at Birmingham, Birmingham, AL, USA

## Abstract

OBJECTIVES/SPECIFIC AIMS: Approximately 50% of people who have been diagnosed with HIV are either not linked to a care provider or not retained in medical care. This has substantial implications for both individual and public health outcomes. On an individual level, being retained in care is necessary for continuous receipt of antiretroviral therapy and sustained viral suppression. The public health implications of poor retention in HIV care are also serious, as it is estimated that people with HIV who are not retained in medical care are responsible for a majority of HIV transmissions, even more than the number of transmissions attributable to those who are HIV infected but undiagnosed. State departments of health routinely collect surveillance data including positive HIV test results, CD4 counts and viral load measures for monitoring trends in HIV infection. A shift in the use of these surveillance measures, guided by the CDC, has brought forth the opportunity to use these data for direct patient services and, more specifically, to direct re-engagement and retention in care efforts. Although the risk factors for poor retention in HIV care have been characterized using information from individual or multiple clinics, this study seeks to incorporate state surveillance data into the retention measures. METHODS/STUDY POPULATION: This retrospective cohort study was performed at the University of Alabama at Birmingham 1917 HIV/AIDS Clinic among patients with at least one attended HIV primary care visit during the calendar year of 2015. Retention during the calendar year of 2016 was then measured as whether or not a patient had 2 or more completed clinic visits which were separated by more than 90 days (in accordance with the Health Resources and Services Administration or HRSA guidelines, a National HIV Quality Indicator). For patients who did not have any primary care visit in 2016, the Alabama Department of Public Health will provide a status of care (out of care, in care elsewhere, died, moved out of state, and cannot locate) based on HIV laboratory results reported from all clinics and labs across the state and/or mortality information. A multinominal regression model of the status of care will be fitted to demographic, clinical, laboratory, and behavioral patient reported outcomes captured during an index visit in 2015. RESULTS/ANTICIPATED RESULTS: Data were recently obtained and is currently being analyzed on 3107 patients included in this study. We anticipate that there will be differences in the factors significantly associated with patients classified as out of care, poorly retained (patients who have only one completed clinic visit), and retained in care by the HRSA measure during calendar year 2016. DISCUSSION/SIGNIFICANCE OF IMPACT: By incorporating state surveillance data into our analysis, we expect to obtain a more precise picture of the risk factors for poor retention among HIV patients. For the first time, we will be able to determine if patients lost to our HIV clinic (~10% annually) are entirely lost to medical care or are seeking care elsewhere as indicated by HIV lab data reported to public health via surveillance. Identified risk factors will then be able to better inform the efforts to proactively improve the efficiency for HIV patient retention and re-engagement, and therefore lead to better individual outcomes for HIV patients and reduce the incidence of new HIV cases.

